# American Dental Association

**Published:** 1886-09

**Authors:** 


### AMERICAN DENTAL ASSOCIATION.

TWENTY-SIXTH ANNUAL MEETING, HELD AT NIAGARA FALLS, AUG. 3, 4, 5 AND 6, 1886.

REPORTED FOR THE INDEPENDENT PRACTITIONER BY "MRS. M. W. J."

The twenty-sixth annual meeting of the American Dental Association was held at Niagara Falls, Aug. 3, 4, 5 and 6, 1886.

The members were called to order at 11 A. M., Tuesday, Aug. 3, the President in the chair.

OFFICERS.

*President*—W. C. Barrett, Buffalo, N. Y.

*First Vice-President*—L. C. Ingersoll, Keokuk, Iowa.

*Second Vice-President*—A. T. Smith, Minneapolis, Minn.

*Recording Secretary*—Geo. H. Cushing, Chicago, Ill.

*Corresponding Secretary*—A. W. Harlan, Chicago, Ill.

*Treasurer*—Geo. W. Keely, Oxford, Ohio.

*Executive Committee*—J. N. Crouse, Chicago, Chairman; A. M. Dudley, Secretary.

*Local Committee of Arrangements*—S. A. Freeman, Buffalo, N. Y.; Geo. L. Field, Detroit, Mich.; J. W. Wassall, Chicago, Ill.

The roll was called, and one hundred and four members found to be present.

The reading of the minutes of the last annual meeting, held in Minneapolis, was dispensed with, they being already published and in the hands of the members.

At that meeting a resolution was offered to amend the constitution in such way as to substitute the 4th Tuesday in August in place of the 1st, as the time of meeting.

The resolution to that effect being the first item of miscellaneous business, was put before the body for discussion. A motion to lay it on the table was carried by a large majority.

Another amendment to the constitution, offered by resolution at the last meeting, putting the selection of place of meeting in the hands of the executive committee, was, on motion of the original mover, indefinitely postponed.

A resolution offered by Dr. A. H. Thompson, Topeka, Kansas, amending Sec. 2 of Art. VI, so as to allow members to join two or more Sections instead of being confined strictly to one, and otherwise altering that section, was, after some discussion, with suggestions of amendments and alterations, laid upon the table temporarily.

*Dr. Crouse*—Said that it was so difficult to get the members to work even one Section, that it was not probable any would wish to join two.

*Dr. Thompson*—Thought it would be beneficial by distributing the few workers among more Sections.

*Dr. J. S. Marshall*—Thought there should be no restrictions upon the amount of work a member might perform. The Sections should be open to all.

*Dr. Geo. H. Cushing*—Called attention to the fact that volunteer papers were referred to the Section to which they pertained, the author having all the privileges of that Section, except in electing its officers. There was no limit to the number of papers, and consequently of Sections, in which any man might work.

The printed programme, subject to such changes as might be deemed necessary, was submitted by the committee of arrangements as a partial report, and on motion, accepted as such.

The committee on publication reported that the transactions of the last annual meeting had been published, as usual, by the S. S. White Co. They had been substantially bound in cloth and mailed to all members in December. The committee expressed their obligations to Mr. Hise for his valuable assistance They also reported that two Mss. copies of the full proceedings of 1883 had been made for the Congressional Library, as per resolution.

An itemized statement of receipts and expenditures was presented, showing the sum of $21.40 due the committee.

The report was received and referred to the Auditing Committee.

The question of indebtedness for back dues was discussed at length, a member having been confronted with a claim for dues of 1875 and 1876, on presenting his credentials as delegate from the Michigan State Society. He stated that he had never been a permanent member; had always paid his dues when present as delegate ; had never been notified of the indebtedness, and had never received any volume of transactions.

Drs. Taft, Allport and others, considered that if the books showed these things to be as stated, there could be no indebtedness on the part of the member.

Dr. Crouse said that local societies could not dictate whom the Association should receive as delegates. That the constitution declared that no delegate could be received with unpaid dues, and that the gentleman not having been accepted as delegate had no right to the floor.

Dr. Taft said that unless the records of the Association showed that the gentleman had declared his intention of becoming a permanent member, there could be no indebtedness.

The subject was, on motion, referred back to the Committee on Credentials, who later in the day reported that the error had arisen from confusion in the books due to transference from one treasurer to another.

Dr. Metcalf was therefore announced as delegate from the State Dental Society of Michigan.

The Treasurer submitted an itemized report showing :

| | |
|---|---|
| Balance on hand Aug. 1885 | $1,948 95 |
| Dues received at Minneapolis | 1,020 00 |
| Additional receipts | 440 00 |
| Total | $3,408 95 |
| Disbursements | 789 85 |
| Balance on hand | $2,619 10 |

This gratifying financial exhibit was greeted with applause.

The President then delivered his annual address, which was received with the applause becoming a scholarly production, and was, on motion of Dr. Dorrance, ordered placed in the hands of the Publication Committee, and spread upon the minutes.

On motion, adjourned to 8 P. M.

Immediately after adjournment the various scientific sections met for organization.

During the afternoon various clinics were held, and appliances exhibited, in the different rooms of the Casino building and at the hotels. The dental depots and manufacturers' exhibits were also open at all times between the sessions.

EVENING SESSION.

The Association was called to order again, by the President, at 8.15 P. M. The minutes of the morning session were read and approved.

Dr. Thompson moved a reconsideration of the resolution amending the article of the constitution having reference to work in sections.

The resolution, which had received some verbal alterations, was again discussed, and finally adopted.

The resolution provides that each member shall join one, and may join two or more Sections; also that at roll-call, on the second day, he shall answer to his name by giving the number of his Section or Sections in place of the customary "here" or "present."

There being no further miscellaneous business, the Sections were called in regular order. The list was gone through with, but not one responded as organized and ready to report.

A motion to adjourn was lost.

The roll of Sections was called a second time.

When Section VII—Physiology and Etiology—was reached, Dr. A. H. Thompson, secretary, reported a list of papers which had been presented to and accepted by the Section. The authors were not ready with their papers. Being last on the list, they had not expected to be called upon first.

The roll of Sections was called again.

Dr. W. B. Ames, secretary of Section I, Prosthetic Dentistry, Chemistry and Metallurgy, reported that the Section had received and approved two papers; one from Dr. W. H. Trueman, on "Recent Improvements in Vulcanizing and Vulcanizers;" and one from Dr. Haskell, on "Needed Improvements in Mineral Teeth." They desired to call attention to a number of valuable papers published in the journals of the past year, and notably, one read before the Kansas Dental Society, and one read before the Connecticut Valley Association. They called attention to the great improvements in carved porcelain blocks, and to the more general use of continuous gum work, due to the improvements in small furnaces, to the skill and ingenuity developed in crown and bridge-work, and to various small appliances used in the mouth, particularly matrices and separators. They commended removable bridge-work, spoke approvingly of a new material for taking impressions, composed of Potter's clay and glycerine, of a new fusible metal composed of sixteen parts bismuth, twenty parts tin and six parts lead. They directed attention to the new methods of Dr. Melotte, on which clinics would be given, and to the continuous gum furnace of Dr. C. H. Land. They requested the consideration of the report before the reading of other papers.

*Dr. Wm. H. Atkinson*—Offered an objection, saying that it would be foisting the work of the Section upon the general body.

*Dr. C. N. Pierce*—Thought the papers should be read first, that the whole subject might be discussed at once, and passed.

The chair decided that the papers should be read previous to the discussion of the report.

Dr. Ames, Secretary of the Section, then read the paper presented by Dr. Trueman. This was a thorough and comprehensive treatise of the principles involved in the process of vulcanizing, and their application in the New-Mode Heater and the Seabury Vulcanizer.

Dr. Haskell's paper described graphically many needed improvements in porcelain teeth, in shapes, size, color, etc.; the changes needed in order to give them individuality of style, it being now found especially difficult to match the natural teeth, where partial plates are required. Many of the styles now made are altogether too small for any but an infant's mouth. Others are so large as to deserve the name of horse-teeth. There is not the proper blending of color in the individual teeth, nor the peculiar gradation of color found in the full set of natural teeth. Variations are needed, too, in the shape and arrangement of the pins, in the thickness and quality of the enamel, etc. It was recommended that a committee be appointed to confer with the leading manufacturers on this subject.

Dr. W. H. Dorrance read a paper in continuation of a former report, giving the tabulated results of wearing plates of the vegetable bases, rubber and celluloid. Leaving out of consideration the results of injuries clearly traceable to uncleanliness, or the mechanical injury from badly made or ill-fitting plates, he found, by these continued observations, that the percentage had increased to fifty-five per cent. In every case radical improvement followed the substitution by metal plates.

Dr. Dorrance read another short paper on Alloys. He gave the formula of a solder of chemically pure metals: silver, one part; zinc, two parts; copper, three parts. This makes a remarkably white alloy, and is used in the proportions of one part of the alloy to two, three or four parts of scrap gold, fusing the gold first. The alloy follows the color of the gold used, whether red or yellow.

Section I. was declared opened for discussion.

*Dr. C. N. Pierce*—Inquired to what the injuries, caused by the plates, were attributed?

*Dr. Dorrance*—Considered them attributable to the retention of animal heat, due to the non-conducting properties of the base. If due to uncleanliness, or badly made plates, the substitution of metal plates would not afford a remedy. He did not consider the coloring matter of rubber a factor.

*Dr. Pierce*—Asked to what he attributed the difference in results from black and red rubber?

*Dr. Dorrance*—Had not found much difference. He had substituted black rubber without benefit.

*Dr. J. G. Friedrichs*—Said that at the rate of fifty-five per cent. named, fifty out of every one hundred people wearing rubber plates would be suffering, in which case there would be ten millions sore mouths in the country, and the facts alleged patent to every practitioner of dentistry, and this he was not prepared to admit. If this were the case the material would not be tolerated by any first-class practitioner, which shows the folly of such conclusions.

*Dr. Louis Ottofy*—Said that the recent investigations of Prof. G. V. Black were very conclusive, and proved that micro-organisms were found under all plates alike, regardless of material.

*Dr. Geo. H. Cushing*—Said that rather misrepresented Dr. Black, whose microscopical investigations proved that micrococci were found under all *unclean* plates, though rubber and celluloid were much more likely to be the home of micrococci than metal.

*Dr. W. H. Morgan*—Said that this Section had insisted, at the last meeting, upon being afforded opportunity for discussion. It was usually crowded out at the close of the session. All that had been said to-night was a mere rehash of what had been repeated for years. It was a mere inference that the mouth eliminates heat, or that the natural heat of the mouth would be destructive to its own tissues. He had hoped that the Section, having had a whole year for preparation, would have presented something worthy of consideration.

*Dr. James Truman*—Said that for a number of years mechanical dentistry had been almost a lost art, and was now in a transition state. He feared that the advocates of bridge-work were going too far. They did not stop to consider the future results of those permanent bands upon the teeth, which are driven home as upon an anvil, impinging upon and irritating a tender, sensitive membrane, violating physiological law and inducing pathological conditions which are not easily overcome. They were going too fast and too far, daily doing work which is liable to result in permanent injury, loosening and destroying teeth. The principles involved should be thoroughly ventilated by the Association.

*Dr. Atkinson*—Expressed his joy in at length hearing one man speak of principles. When bold statements are made, however, it was essential to have a strong foundation of facts. It was a mistake to say that the pericementum was irritable and tender. If there was one tissue in the human body that would bear abuse, it was connective tissue.

The whole discussion revealed lack of knowledge of principles. Crowns and bridge-work were inserted without inquiring as to how much loss there was at the end of the root, or how much softening around it. Bridge-work, when properly constructed and inserted, is the *ne plus ultra* of replacement.

*Dr. F. H. Rehwinkle*—Announced that the Central German Association of dentists was now in session, at Franfort-on-the-Main, and moved that a telegram of fraternal greeting be sent, signed by the officers of the Association.

Carried.

Dr. Geo. H. Cushing had a communication from the Librarian of the Patent Office Reading Rooms, London, requesting copies of the Proceedings of the Association, for the benefit of some two hundred daily readers, men especially interested in scientific patents and inventions.

On motion of Dr. Taft, the Secretary was instructed to comply with the request, by forwarding as complete a set of Proceedings as possible.

On motion, adjourned to 9 A. M.

(TO BE CONTINUED.)

